# Different Regulatory Strategies of Arsenite Oxidation by Two Isolated *Thermus tengchongensis* Strains From Hot Springs

**DOI:** 10.3389/fmicb.2022.817891

**Published:** 2022-03-11

**Authors:** Changguo Yuan, Ping Li, Chun Qing, Zhu Kou, Helin Wang

**Affiliations:** ^1^State Key Laboratory of Biogeology and Environmental Geology, China University of Geosciences, Wuhan, China; ^2^Hubei Key Laboratory of Yangtze Catchment Environmental Aquatic Science, China University of Geosciences, Wuhan, China

**Keywords:** *Thermus*, arsenite oxidation, regulatory strategy, RT-qPCR, thermophiles

## Abstract

Arsenic is a ubiquitous constituent in geothermal fluids. Thermophiles represented by *Thermus* play vital roles in its transformation in geothermal fluids. In this study, two *Thermus tengchongensis* strains, named as 15Y and 15W, were isolated from arsenic-rich geothermal springs and found different arsenite oxidation behaviors with different oxidation strategies. Arsenite oxidation of both strains occurred at different growth stages, and two enzyme-catalyzed reaction kinetic models were observed. The arsenite oxidase of *Thermus* strain 15W performed better oxidation activity, exhibiting typical Michaelis–Menten kinetics. The kinetic parameter of arsenite oxidation in whole cell showed a *V*_*max*_ of 18.48 μM min^–1^ and *K*_*M*_ of 343 μM. Both of them possessed the arsenite oxidase-coding genes *aioB* and *aioA*. However, the expression of gene *aioBA* was constitutive in strain 15W, whereas it was induced by arsenite in strain 15Y. Furthermore, strain 15Y harbored an intact *aio* operon including the regulatory gene of the ArsR family, whereas a genetic inversion of an around 128-kbp fragment produced the inactivation of this regulator in strain 15W, leading to the constitutive expression of *aioBA* genes. This study provides a valuable insight into the adaption of thermophiles to extreme environments.

## Introduction

Arsenic (As) is extremely poisonous to humans and other living organisms. However, it is widely distributed in many natural and artificial ecosystems, including geothermal springs (Hug et al., 2017; Mukherjee et al., 2017). Due to its toxicity, the World Health Organization has classified As as a carcinogenic pollutant and set its exposure limit to below 10 μg l^–1^ (Duoc et al., 2020). Over 100 tons of As is discharged every year from geothermal fluids into soils, river streams, and groundwater, thus posing a significant threat to the environment (Bundschuh and Maity, 2015; Wang et al., 2018). The bioavailability and physicochemical properties of As are closely related to the chemical form of As (Kumarathilaka et al., 2018). As species have four valence states: arsine (−3), elemental As (0), arsenite (As[III]) (++3), and arsenate (As[V]) (+5). Of these, As[III] and As[V] are predominant forms in natural aquatic environments, with As[III] being more toxic and mobile than As[V] (Oremland and Stolz, 2003).

Microorganisms have evolved multiple strategies to adapt to As-contaminated environments, including oxidation, reduction, and methylation of As. The first known bacterial strain capable of oxidizing As[III] was isolated in 1919 (Green, 1919); since then, dozens of phylogenetically diverse As[III] oxidizers have been isolated from various As-rich environments. As[III] oxidase (Aio) is an ancient bioenergetics heterodimeric enzyme that comprises two subunits: a large, molybdopterin-containing subunit and a smaller Rieske subunit (Warelow et al., 2017). Many of these isolates are chemolithoautotrophic and grow using As[III] as an electron donor, whereas other heterotrophic strains oxidize As[III] as part of a detoxification process. The molecular and genetic bases of As[III] oxidation are relatively well established (Andres and Bertin, 2016). There are various regulatory models of microbial As[III] oxidation, including three-component signal transduction system AioXRS and an ArsR/SmtB family regulator AioF with AioRS and a phosphate transport two-component system PhoBR (Shi et al., 2020).

Thermophiles are characterized by deep phylogenetic trees with short branches, suggesting that several metabolic pathways may have been derived from these ancient genera (Vaidya et al., 2018). Organisms of the genus *Thermus* are typically of the thermophilic group and are distributed globally across hot spring environments (Brock and Freeze, 1969). Genome sequencing analysis has revealed that *Thermus* species have highly versatile carbon metabolic pathways and that they perform incomplete denitrification (NO_3_^–^→N_2_O) and metabolize sulfur (Murugapiran et al., 2013). Our previous study revealed that members of this genus are also involved in rapid As[III] oxidation within geothermal fluids (Jiang et al., 2016). Furthermore, both reductive and oxidative metabolic activities have been observed in *Thermus* species under anoxic and oxic conditions, respectively (Gihring et al., 2001). Two kinds of key genes associated with redox functions have been identified in *Thermus* species: *aioBA* and *arsC* (Del Giudice et al., 2013). The regulation of As[V] reduction and ArsC enzyme properties has been well studied (Antonucci et al., 2017). However, the physiological and biochemical characteristics of As[III] oxidation in the genus *Thermus*, as well as its molecular regulation, remain poorly understood.

In the present study, two *Thermus tengchongensis* strains were isolated from geothermal fluids and found to have different As[III] oxidization behaviors. We hypothesized that the distinct microbial As[III]-oxidization behaviors in two strains may be linked to the regulation of the *aioBA* genes found in both strains. For this, we measured their gene expression levels and analyzed the *aio* operon gene structure. To our knowledge, this is the first study to focus on the As[III] oxidation process at the gene expression level in various *Thermus* strains.

## Materials and Methods

### Study Site and Sample Collection

Water samples were collected from the Hamazui site (24°56′59″N, 98°26′17″E), located in Rehai geothermal area, Tengchong region, Yunnan province, China. These geothermal fluid samples were characterized for total As concentrations (197.3∼321.1 μg l^–1^), extremely high temperature (82.5∼91.8°C), and slightly alkaline pH (7.4∼10) (Guo et al., 2017). Water samples (1 l) were filtered through 0.22-μm membrane filters (Millipore, Bedford, MA, United States) to collect microbial biomass. The filtered membranes were stored in 50-ml sterile polypropylene tubes at 4°C before enrichment.

### Enrichment and Isolation of As[III]-Oxidizing Strains

The filtered membranes were cut into pieces and then inoculated in a 100-ml enrichment medium (TYL, pH = 8.8) with 1 mM As[III], which consisted of (per liter) 0.25 g tryptone, 0.25 g yeast extract, 30 mM sodium lactate, and 1 ml 100 × stock solution, as listed in the following (in 100 ml): 1 g citric acid, 3.8 g KCl, 3.6 g NaCl, 0.2 g CaSO_4_⋅2H_2_O, 1 g MgSO_4_⋅7H_2_O, 1.11 g Na_2_HPO_4_, 0.5 g FeCl_3_, and 5 ml trace element solution, which included 0.5 ml l^–1^ concentrated sulfuric acid, 2.28 g l^–1^ MnSO_4_⋅H_2_O, 0.5 g l^–1^ ZnSO_4_⋅7H_2_O, 0.5 g l^–1^ H_3_BO_3_, 0.025 g l^–1^ CuSO_4_⋅5H_2_O, 0.025 g l^–1^ Na_2_MoO_4_⋅2H_2_O, and 0.045 g l^–1^ CoCl_2_⋅6H_2_O. Enrichments were cultivated for 5 days while shaking on a rotary shaker (120 rpm) at 60°C in aerobic conditions.

To isolate As[III] oxidizing strains, the enrichments were serially diluted (10-fold) and 100-μl aliquot cultures with 10^–7^ dilution were spread on growth medium containing 1 mM As[III]. The inoculated agar plates were incubated at 60°C, and colonies were picked. To obtain pure strains, isolates were repeatedly subcultured on TYL medium. The As[III]-oxidizing isolates were first screened out by the KMnO_4_ method in TYL liquid medium (Bahar et al., 2020). The specific operation was modified as follows: 200 μl of bacterial suspension was added into 96-well plates and incubated for 48 h at 60°C, then mixed with 10 μl of 10 mM KMnO_4_. A pink color mixture indicated As[III] oxidation. The theoretical positive strains were preserved in 20% glycerol at −80°C for further experiments.

### Phenotypic and Physiological Characterization

Colony morphology and pigmentation were determined on TYL agar plates at 60°C. Cell morphology was observed using a fluorescence microscope (Leica, Wetzlar, Germany). The ranges of temperature, pH, and salinity were determined at different temperatures (40, 50, 60, 70, and 80°C), pH (5, 6, 7, 8, 9, and 10), and salinities (0, 0.5, 1, and 2%) using TYL medium. MICs of As were determined by growing the strains in TYL medium with different concentrations of NaAsO_2_ and Na_3_AsO_4_⋅12H_2_O.

### As[III] Oxidation Assay

Cells were grown to late exponential phase in TYL medium (100 ml) under 60°C. A series of batch experiments were designed to determine the ability of As[III] oxidation with long-term and short-term responses under aerobic conditions. For long-term response, suspension cultures (1 ml) containing about 10^7^ CFU were inoculated into fresh TYL medium (100 ml) containing 1 mM As[III]. For short-term response, when cells were inoculated into fresh medium, 1 mM As[III] was not added until the cultures were in the stationary phase. Bacterial suspensions (1 ml) were taken for measurements of optical density at OD 660 nm and As[III]/As(V) concentrations. To estimate the As[III] oxidation rate quantitatively, a kinetics study was conducted under aerobic conditions. For this purpose, strains grown at the late exponential phase without As[III] were harvested by centrifugation at 8,000 g for 20 min at 4°C. Cells were washed twice with PBS buffer (pH 7.2) and resuspended into 1 ml fresh medium to obtain 10^7^ CFU ml^–1^. Oxidation started by adding 0.2, 0.4, 0.8, 1.6, 2, 3, 4, and 5 μl of As[III] stock solution (500 mM). Control was set without cells, and samples were collected with a 25-min interval for As species determination.

As speciation was determined by liquid chromatography–hydride generation–atomic fluorescence spectrometry (LC-HG-AFS, Haiguang, AFS-9780, Beijing) (Jiang et al., 2015). Controls without inoculation were kept under the same condition, and all tests were performed in 250-ml conical flasks. All values are expressed as a mean of three replicates with ± standard deviation (SD). The data were analyzed by OriginPro 2016.

### Phylogenetic Analyses Based on 16S rRNA Gene and AioA

To identify the phylogenetic relationship of strains 15Y and 15W, DNA was extracted using the SDS method as described previously (Wright et al., 2017). The 16S rRNA gene was amplified by PCR using Bac27F/Univ1492R primers described in Supplementary Table 2 (Jiang et al., 2006), and PCR products were sent to Shanghai Shenggong Bioengineering Company for sequencing. Illumina sequencing was performed using the commercial service of Novogene (Beijing). Genome annotation and bioinformatics analysis were performed as described previously (Jia et al., 2017). DNA–DNA hybridization (DDH) was calculated by a Genome-to-Genome Distance Calculator (Auch et al., 2010). AioA amino acid sequences were inferred from *aioA* gene sequences extracted from genome sequences. Phylogenetic trees inferred from sequences of 16S rRNA gene and putative AioA were constructed using MEGA7 (Kumar et al., 2016).

### RNA Extraction and *aioBA* Gene Expression for Short-Term Response

To compare the *aioBA* gene expression of two strains for short-term response to As exposure, batch tests were performed in three biological replicates with 1-mM As[III] treatment for the whole As[III] oxidation process. Two strains were cultured under 60°C in TYL medium. When they grew to stationary phase, cells were resuspended into fresh medium and incubated with 1 mM As[III]. The incubation time points included 0.5, 2, 4, and 6 h. Cells were centrifuged at 8,000 g for 20 min at 4°C for RNA preparation. Total RNA was extracted using TRIzol reagent (Invitrogen, Carlsbad, CA, United States) following the manufacturer’s instructions with some modifications. Cells were treated with 250 μl lysozyme (20 mg l^–1^) before RNA extraction. The RNA samples were treated with PrimeScript RT Reagent Kit with gDNA Eraser (TaKaRa) for 2 min at 42°C to remove genomic DNA. RNA was checked on agarose (2%) gel electrophoresis, and the concentration was determined using a NanoDrop spectrophotometer (Thermo Fisher Scientific, Gene Company Ltd., Waltham, MA, United States) and diluted to equal concentrations. DNA-free RNA (1 μg) was used for cDNA synthesis according to the manufacturer’s instructions. Each reaction mixture (20 μl) consisted of 4 μl RNase-free H_2_O, 4 μl 5 × PrimeScript buffer, 1 μl RT Primer Mix, 1 μl PrimeScript Enzyme Mix, and 10 μl DNase-treated RNA sample. Reactions were incubated at 37°C for 15 min followed by heat inactivation 85°C for 15 s.

The RT-qPCR was performed with ABI QuantStudio 3 Real-Time System (Applied Biosystems, Foster City, CA, United States) using qPCR kit (TB Green™ Premix Ex Taq™ II, TaKaRa). Each reaction mixture contained 10 μl TB Green Premix Ex Taq II, 0.8 μl forward and reverse specific primers, 0.4 μl ROX Reference Dye II, 6 μl RNase-free water, and 2 μl diluted template (cDNA, non-reverse transcription RNA, or water). The housekeeping gene, encoding ATP-dependent DNA helicase-*recG*, was used as an endogenous control to normalize the cycle threshold (Ct) value (Ratner et al., 2019). Two As[III] oxidation genes (*aioA* and *aioB*) and two internal genes (*recG* and 16S rRNA) were quantified. Melting curve analysis was performed to check the specificity of amplification. The relative expression was calculated according to the 2^–△△Ct^ method (Livak and Schmittgen, 2001). Meanwhile, RT-PCR was done to detect the expression of the abovementioned genes for qualitative analysis. Software Primer 5.0 was used to design primer sequences of target genes. Related primer sequence and amplification programs in this study are shown in Supplementary Table 2.

### The Comparison of As[III] Oxidase Gene Clusters and Promoter Sequence Analysis

The *aio* gene clusters of strains 15Y and 15W were analyzed by IBS software (Liu et al., 2015). Besides the comparison of various *Thermus* species, typical *aio* gene clusters of representative As[III] oxidizers from other environments were also used to observe their discrepancy with the genus *Thermus*.

A promoter prediction program, BPROM (Salamov and Solovyevand, 2011), was used to analyze the sequence upstream of *aioB* (www.softberry.com). The transcription start site was predicted using the Neural Network Promoter Prediction from the Berkeley Drosophila Genome Project (Reese, 2001). The upstream region (201 bp) of *aioB* was scanned for putative binding sites motifs using XSTREME (Grant and Bailey, 2021).

## Results and Discussion

### Morphological and Physiological Characterization

Two *Thermus* strains designated as 15Y and 15W were isolated from a hot spring. Both strains were rod-shaped, gram-negative, heterotrophic, and thermophilic. They produced different-colored colonies (yellow and pale yellow, respectively) when grown on TYL agar plates (Supplementary Figure 1). The growth ranges for 15Y and 15W were 55-75°C, pH 6.0-10.0, and 0-1% NaCl (w/v). The morphological and physiological characterizations were similar with a previous isolated strain of *Thermus* (Yu et al., 2013). Different maximum inhibitory concentrations (MICs) of As were observed for each strain. Strain 15Y exhibited an MIC of 4 mM for As (III) and 25 mM for As(V), whereas the respective MICs for strain 15W were 3 and 8 mM. Strain 15W had a more pronounced lag phase in its growth curve compared to strain 15Y and aggregated into visible particles during its early growth stages. Upon exposure to 1 mM As[III], the lag phase of strain 15Y increased, whereas strain 15W was less affected by As[III] throughout its entire growth stage (Supplementary Figure 2). This is the amount of time strain 15Y needed for its As[III]-oxidation response to become fully engaged. The aggregated particles and extension of the lag period might be a strategy of As resistance.

### Phylogenetic Analysis of As[III] Oxidizers

BLAST analysis of the 16S rRNA gene sequences of strains 15Y (1,548 bp) and 15W (1,552 bp) revealed that two strains belonged to the previously described species *T. tengchongensis* (Yu et al., 2013), with sequence similarities of 99.74 and 99.48%, respectively. The sequence similarity between the 16S rRNA gene of two strains was 99.4%. The average nucleotide identities between two isolates and *T. tengchongensis* YIM 77401 (GenBank no. NZ_JQLK00000000) were 98.55 for 15Y and 97.88% for 15W (>95%), respectively, according to MUMmer alignment^1^. The DDH between the two bacterial genomes was estimated to be approximately 88.5% (>70%). The genomic characteristics of 15Y and 15W were similar with each other, such as 2.4 Mb for genomic size, 21 and 22 fragments for interspersed repeat, and 98 and 108 fragments for tandem repeat, respectively. Notably, strain 15W had more genes for transposases (16 vs. 6). Other genome information of the two isolates and the closely related reference strain is listed in detail in Supplementary Table 1. A phylogenetic dendrogram was then constructed based on the 16S rRNA gene sequences of the isolated strains and those of other known As[III]-oxidizing isolates retrieved from GenBank (Supplementary Figure 3). As[III] oxidizers were widely distributed across Proteobacteria (Santini et al., 2000; Muller et al., 2006; Hoeft et al., 2007; Zhang et al., 2015), Firmicutes (Wu et al., 2017), and Deinococcus–Thermus (Gihring and Banfield, 2001). As expected, strains 15Y and 15W clustered with the other *Thermus* species, forming a clade separated from the other genera (Supplementary Figure 3). Our results suggested that two strains belong to the species *T. tengchongensis*.

**FIGURE 1:**
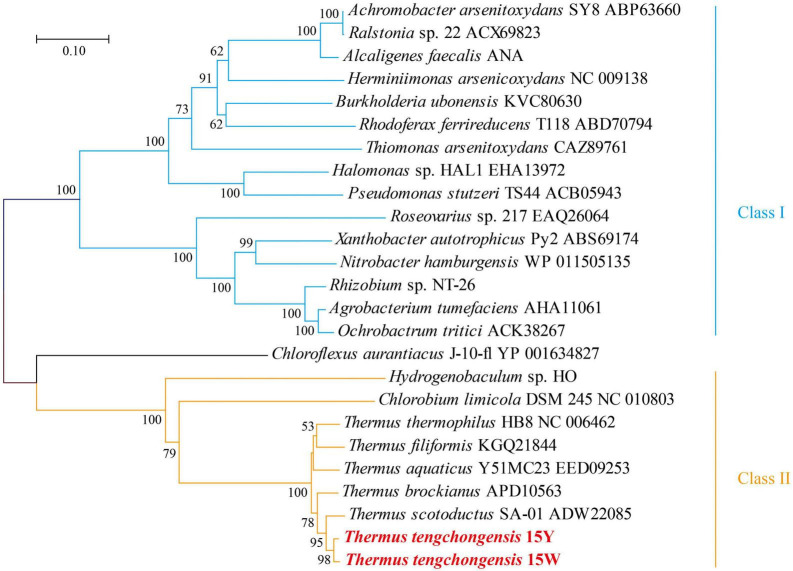
Phylogenetic neighbor-joining dendrogram inferred from deduced amino acid sequences of AioA of strains 15Y and 15W with known representative As[III] oxidizers. The stability of the relationship was assessed by bootstrap values (shown > 50 for 1,000 resampling) for the tree topology of neighbor-joining data. Bars at the bottom of the tree denote the percentage of substitutions.

The sequence similarities of the Aio protein large subunit (AioA) between strains 15Y and 15W and *T. tengchongensis* YIM 77401 were 100% and 98.7%. AioA sequence similarities were high when compared to other *Thermus* species (>91.2%), but low when compared to other genera, such as *Herminiimonas arsenicoxydans* ULPAs-1 (38.0-38.6%), *Rhizobium* sp. NT-26 (37.5%-37.9%), and *Agrobacterium tumefaciens* strain 52 (38.2-38.4%) (Figure 1). According to the phylogenetic analysis, all translated amino acid sequences of AioA were classified into two clusters. Cluster I comprised β-/γ-Proteobacteria and α-Proteobacteria, respectively. Even though some members of this cluster also have low similarity, they share common regulatory gene of As[III] oxidation, which is characterized by AioRS. The regulatory mechanisms of As[III] oxidation in Cluster I have been relatively well studied, whereas those of Cluster II remain comparatively unclear. Our results suggest that members of the genus *Thermus* also have a relatively distant phylogenetic relationship with other As[III] oxidizers. Therefore, we speculated that *Thermus* strains might use very different regulatory strategies to cope with As[III] stress.

### Comparison of as Oxidation Behaviors Between Two Strains

**FIGURE 2:**
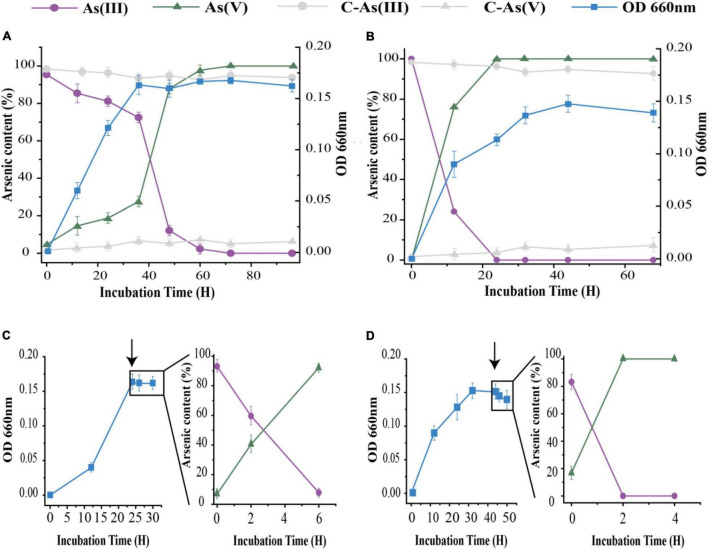
As[III] oxidation by strains 15Y **(A,C)** and 15W **(B,D)** under aerobic condition with 1 mM As[III]. **(A,B)** represent the data in long-term response, **(C,D)** in short-term response. Black arrows represent the time point of As[III] addition. Error bars indicate the standard deviation (*n* = 3).

Under aerobic conditions, both strains could oxidize As[III] to As(V) as part of both long- and short-term responses (Figure 2). We first tested the long-term As[III] responses of the isolates. More than 99% of 1 mM As[III] was oxidized within 60 h by strain 15Y, in which the majority of oxidizing activity occurred between the late log phase and the early stationary phase (20-60 h; Figure 2A). Strain 15W was able to oxidize the same amount of As[III] in < 24 h; As[III] oxidation by 15W occurred during the log phase (Figure 2B). Only a small amount of As[III] (<5%) was oxidized in the abiotic control, indicating that As[III] oxidation occurred through biological means (Figure 2). To further characterize the differences in oxidation rates between the two strains, we then conducted short-term As[III] response experiments, in which As[III] was not added to the medium until the cultures had entered a stationary phase. Strain 15Y required 6 h to completely oxidize 1 mM As[III] (Figure 2C), whereas strain 15W could quickly oxidize the same amount of As[III] in 2 h (Figure 2D).

The kinetic rate constants of As[III] oxidation for two strains were determined based on the whole-cell system. This could truly reflect their own kinetic characteristics of As[III] oxidation. An examination of the reaction kinetics of As[III] oxidation indicated that the two strains exhibited different relationships between the initial As[III] concentration and reaction rates (Supplementary Figure 4 and Supplementary Table 3). Strain 15W conformed to typical Michaelis–Menten kinetics, exhibiting *V*_*max*_ and *K*_*M*_ constants for the Aio enzyme of 18.48 μM min^–1^ and 343 μM, respectively. Some As[III] oxidizers have lower *K*_*M*_ values than *Thermus* strains (343 μM), e.g., *Variovorax* sp. MM-1 (17 μM), which indicates that they have a higher affinity for As[III]. However, strain 15Y showed the highest *V*_*max*_ based on previous literature reports regarding kinetic parameters for As[III] oxidation (Bahar et al., 2013). Furthermore, the rapid As[III] oxidation property was also observed in other *Thermus* species (Gihring et al., 2001). Strain 15W with a rapid As[III]-oxidizing capacity would have an application prospect in As removal. Conversely, the kinetic model for Aio activity in strain 15Y was different with strain 15W. It was not a typical Michaelis–Menten model. Within the range of measurement, under approximately 650 μM As[III], the oxidation rate reached its lowest point, after which the reaction rate began to increase as the As[III] concentration increased between 650 and 2,000 μM. However, the oxidation rate showed a decreasing trend over 2 mM of As concentration. A possible explanation is that the initial slow rate of oxidation in 15Y could be because the oxidation rates were mainly influenced by cell viability when the initial concentrations of As[III] were below 650 μM, during which metabolic activity was inhibited with increasing As[III] concentrations. Strain 15Y likely exhibited higher oxidation rates at lower As[III] concentrations due to general adaptive mechanisms to endure As[III] stress, as the lower As[III] concentrations were comparable to *in situ* geothermal conditions (1-10 μM). However, when initial As[III] concentrations were > 700 μM, oxidation activity increased with As[III] concentration, possibly due to the shortened response time of *aio* operon regulation. After more than 2 mM, cell metabolism was significantly inhibited by As[III] toxicity. Compared with strain 15W, strain 15Y exhibited a slower oxidation rate, up to 3 μM min^–1^. The different As[III] oxidation models between two strains suggested that they employed different As[III] oxidation strategies to cope with As[III] stress. In summary, cells of strain 15Y would make different responses depending on the As[III] concentration. However, the reason why strain 15Y exhibited a special As[III] oxidation model needs to be further studied.

### Transcriptional Analysis of As[III] Oxidase Gene Expression

**FIGURE 3:**
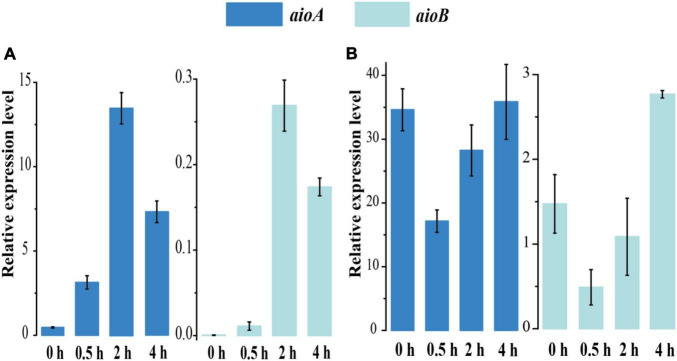
RT-PCR results of *aioAB* gene relative expression levels of strains 15Y **(A)** and 15W **(B)** with 1 mM As[III] treatment following the time 0 to 4 h.

To explore the effect of As[III] treatment on the gene expression of *aioBA*, we monitored the dynamic expression level at various time points under 1 mM As[III] treatment using quantitative real-time PCR (RT-qPCR) (Figure 3). The *aioA* gene expression levels of strain 15Y were increased over the course of As[III] treatment, indicating that the *aioA* gene expression was strongly induced by As[III] (Figure 3A). The peak of *aioA* and *aioB* expression occurred at 2 h in strain 15Y, matching the As[III] oxidation curve associated with the short-term response. By contrast, the expressions of *aioB*/*A* were initially relatively high in strain 15W and subsequently decreased following the addition of As[III]. The *aioB*/*A* expression level did not return to their original state until As[III] was completely oxidized (Figure 3B), which might have been a result of the metabolic burden afforded by 1 mM As[III].

Reverse transcription-PCR (RT-PCR) was used as another approach to verify the apparent constitutive expression of *aioA* in strain 15W (Supplementary Figure 5). RNA samples were collected from strains 15Y and 15W after treatment with 1 mM As[III] at various time points (0, 0.5, 2, and 4 h). Two genes, *aioA* and 16S rRNA gene (as a control), were amplified to produce the fragments with the predicted sizes after cDNA was synthesized. A band matching the fragment size of *aioA* was present in strain 15W before the cells were treated with 1 mM As[III] (Supplementary Figure 5B). By contrast, no PCR product was obtained for strain 15Y prior to As[III] treatment (Supplementary Figure 5A). This suggested that As[III] oxidation could be attributed to *aioBA* expression in both strains; however, their oxidation strategies were different, namely, constitutive type for strain 15W and inducible type for strain 15Y.

### As[III] Oxidase Gene Characteristics in *Thermus* Strains

**FIGURE 4:**
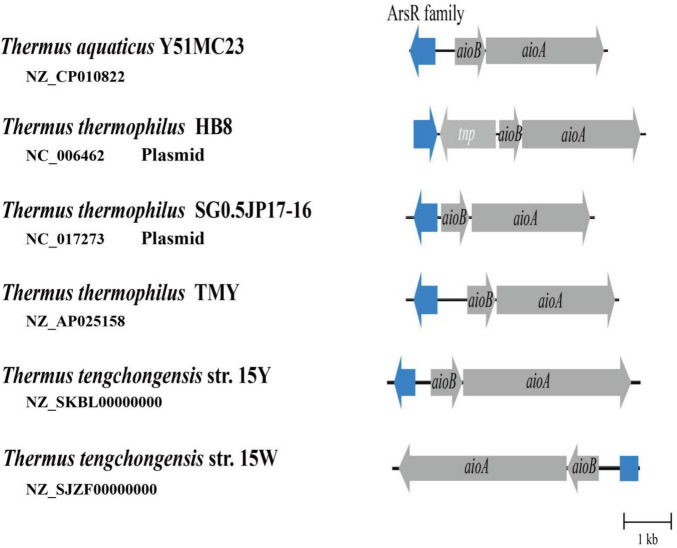
As[III] oxidase gene clusters from multiple *Thermus* strains. *aioA*, As[III] oxidase large subunit gene; *aioB*, As[III] oxidase small subunit gene; *tnp*, transposase. Blue color represents ArsR family transcriptional regulatory gene or its defective gene sequence.

Based on genomic data acquired from the NCBI database, *aioA* was found to be widely distributed among members of the genus *Thermus*. Although the AioA sequence similarity for strains 15Y and 15W was 98.7%, genome annotation results indicated that the *aio* gene clusters were different. Compared with strain 15Y, strain 15W lacks a complete ArsR family regulatory gene, due to this gene being truncated as determined by sequence alignment (Figure 4). The ArsR family is a trans-acting repressor with other potential regulatory functions (Kang et al., 2016; Rawle et al., 2021). The absence of regulatory function in strain 15W could explain why the two strains exhibited differences in As[III] oxidation (Nei et al., 2010). To our best knowledge, all isolated *Thermus* strains bearing *aioBA* genes except for 15W have regulatory genes upstream of the *aio* operon by analyzing existing genomes. Even though ArsR family members have been reported in the regulation of As oxidation in different taxa, they also contain a AioRS two-component system at the upstream of *aioBA* (Moinier et al., 2014; Rawle et al., 2021). Besides AioRS, AioX, a periplasmic As[III]-binding protein, can also regulate As[III] oxidation (Liu et al., 2012). This is significantly different from the single-component system which only existed in *Thermus* so far. The role of the ArsR family needs to elucidated in future research. The different *aio* operons in two strains play an important role in their *aioBA* gene expression.

**FIGURE 5:**
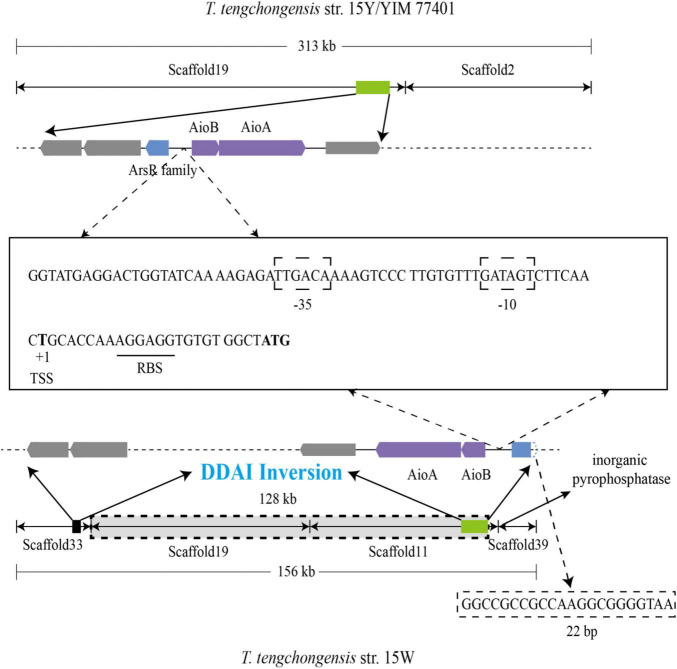
Large-scale inversion through DNA duplication associated with inversion (DDAI) in strain 15W and sequence analysis of *aio* promoters between the ArsR family gene and *aioB* from *T. tengchongensis* strains 15Y (234 bp), 15W (234 bp), and YIM 77401 (201 bp). TSS, transcription start site, and RBS, ribosome binding site, were also shown. The predicted –35 and –10 boxes between two genetic elements are boxed, and the RBS is underlined.

To explore the reason why the ArsR regulatory gene in strain 15W is incomplete, we aligned and analyzed large fragments from three strains of *T. tengchongensis*: 15Y, 15W, and YIM 77401. Sequence alignment analysis using YIM 77401 as a reference genome showed large-scale chromosomal inversion (∼128 kb) in strain 15W (Figure 5). One breakpoint of this genomic mutation event occurred within the first 22 bp of the ArsR family regulatory genes, leading to the destruction of the ArsR family regulatory gene structure; there was an extra gene producing inorganic pyrophosphatase near another breakpoint located at scaffold 39, which catalyzes the hydrolysis of inorganic pyrophosphate to orthophosphate. The birth and death of genes due to chromosomal inversion events have also been documented in *Helicobacter pylori* and *Thiomonas* spp., which is found in the human stomach. In the present study, discrepancies in molecular mechanisms among four inversions reflected the large-scale inversion that occurred in strain 15W, as a DNA duplication associated with inversion (DDAI) (Arsène-Ploetze et al., 2010; Furuta et al., 2011). DDAIs have been identified in various organisms (Kobayashi, 2016) and are a driving force of bacterial evolution (Merrikh and Merrikh, 2018).

Intriguingly, 234 base pairs were identical between *aioB* and *arsR* family members in strains 15Y and 15W, indicating that high conservation of this sequence is essential for the transcription of up- and downstream genes (Westmann et al., 2018). Consistently, the conserved sequence with only 201 bases was also observed in type strain YIM 77401 of *T tengchongensis*. The similarity of three sequences of 201 bp from three strains is 100%. To detect a promoter, we analyzed the sequence upstream of *aioB* and predicted boxes binding to RNA polymerase at positions −35 (TTGACA) and −10 (GATAGT). We also predicted a transcription start site (thymine site, +1) and a ribosome-binding site (Figure 5). 5 putative binding motif sites were observed between the ArsR family gene and *aioB*, including motif1 (5′-ACAAAAGTCCCTTG-3′), motif2 (5′-GTGAGGG-3′), motif3 (5′-ATGAGGACTGGTA-3′), motif4 (5′-GGAAAGGATGC-3′), motif5 (5′-CAGGCTATACTTTA-3′). However, further studies are needed to verify these binding sites. These demonstrated that the integrity of the promoter might be necessary for *aioBA* transcription in *T. tengchongensis*.

### Environmental Adaption of Thermophiles

As-rich geothermal fluid is an extreme environment, in which microorganisms play vital roles in As transformation. The intense conditions of geothermal fluids have influenced the metabolisms of the microorganisms living within them (Crognale et al., 2017). The genus *Thermus*, a predominant inhabitant of geothermal fluids, is involved in multiple elemental biogeochemical cycles. To adapt to extreme conditions, such as high temperature and heavy metal stress, these bacteria have evolved unique metabolic and regulatory mechanisms. A chromosomal inversion in the ArsR family regulatory gene likely provided strain 15W with an evolutionary advantage for inhabiting the As-rich geothermal environment within a limited As concentration range. Similar genetic mutations have been found in other microbes, which are thought to be adaptations for survival, such as mutator phenotypes and adaptive mutations (Okamoto et al., 2007). There are many cases that microorganisms adapt to the changing environment through genomic evolution (Allen et al., 2009; Santos-Beneit, 2015). Mutations in As-related genes could have improved the chances of survival in As-rich environments.

Previous studies have suggested that more primitive two-gene operons have played important roles in the early stages of life, enabling microbes to colonize extreme ecological niches (Rosenberg, 2001). The evolution of the regulatory strategy might have reduced energy consumption, thus reserving energy to cope with the extreme thermal and As environments (Giraud et al., 2001; Kussell et al., 2005). It is well established that prokaryotes play a vital role in the environmental As transformation and high As environments also shape the regulatory model in bacteria (Hamamura et al., 2009). This study provides valuable insight into the adaptive evolution of microorganisms in As-rich geothermal environments.

## Conclusion

In this study, we identified two different regulatory strategies of As[III] oxidation from multiple aspects. The *aioBA* gene set of strain 15Y was induced with the presence of As[III], whereas the gene expression of *aioBA* in strain 15W belonged to the constitutive type. A large fragment inversion occurred in strain 15W, which inactivated the function of the regulatory gene, which might results from a special strategy for coping with As[III]. To our knowledge, we found that this is the first constitutive As[III] oxidation strain and clearly explained in terms of physiology and gene expression. This work provides evidence that thermophiles would respond to environmental changes through genome evolution and gene gain or loss, to increase the possibility of survival in extreme environments.

## Data Availability Statement

The datasets presented in this study can be found in online repositories. The names of the repository/repositories and accession number(s) can be found below: https://www.ncbi.nlm.nih.gov/genbank/, MN477898; www.ncbi.nlm.nih.gov/genbank/, MN480479; www.ncbi.nlm.nih.gov/genbank/, NZ_SKBL00000000; and https://www.ncbi.nlm.nih.gov/genbank/, NZ_SJZF00000000.

## Author Contributions

CY was responsible for the design, execution of the study, and wrote the original manuscript. PL carried out reviewing and editing manuscript and study supervision. CQ, ZK, and HW assisted sampling for experiments. All authors read and approved the final manuscript.

## Conflict of Interest

The authors declare that the research was conducted in the absence of any commercial or financial relationships that could be construed as a potential conflict of interest.

## Publisher’s Note

All claims expressed in this article are solely those of the authors and do not necessarily represent those of their affiliated organizations, or those of the publisher, the editors and the reviewers. Any product that may be evaluated in this article, or claim that may be made by its manufacturer, is not guaranteed or endorsed by the publisher.

## Footnotes

1
http://jspecies.ribohost.com/jspeciesws
