# Supplementary material for: Different Regulatory Strategies of Arsenite Oxidation by Two Isolated Thermus tengchongensis Strains From Hot Springs
Source: Front Microbiol. 2022 Mar 11;13:817891. doi: 10.3389/fmicb.2022.817891 (PMC8963470; doi:10.3389/fmicb.2022.817891)
Supplement: Supplementary file 1 [file Data_Sheet_1.docx]

Supplementary Material

**Supplementary Figures and Tables**

**Supplementary Figures**


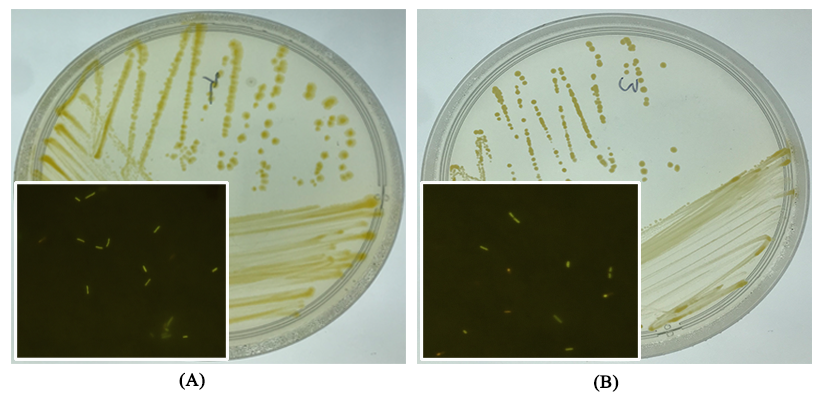


**Supplementary Figure 1.** Morphological of colony and fluorescence microscopy images in the TYL agar plate both strains 15Y (A) and 15W (B).

**
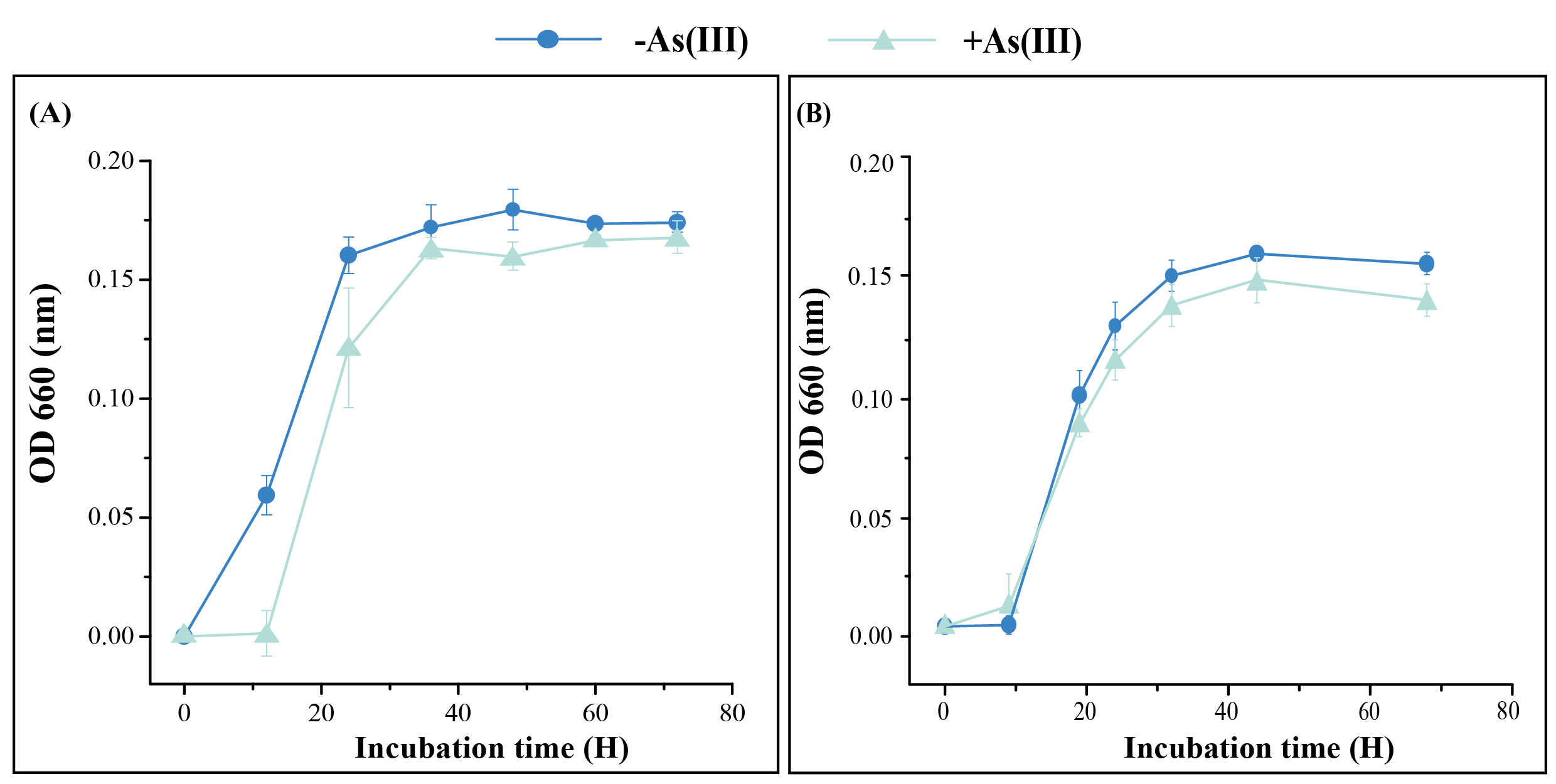
**

**Supplementary Figure 2.** Bacterial growth curves of strains 15Y(A) and 15W(B) with 1 mM As[III] and control (without As[III] ). Error bars indicate the standard deviation (n=2).

**
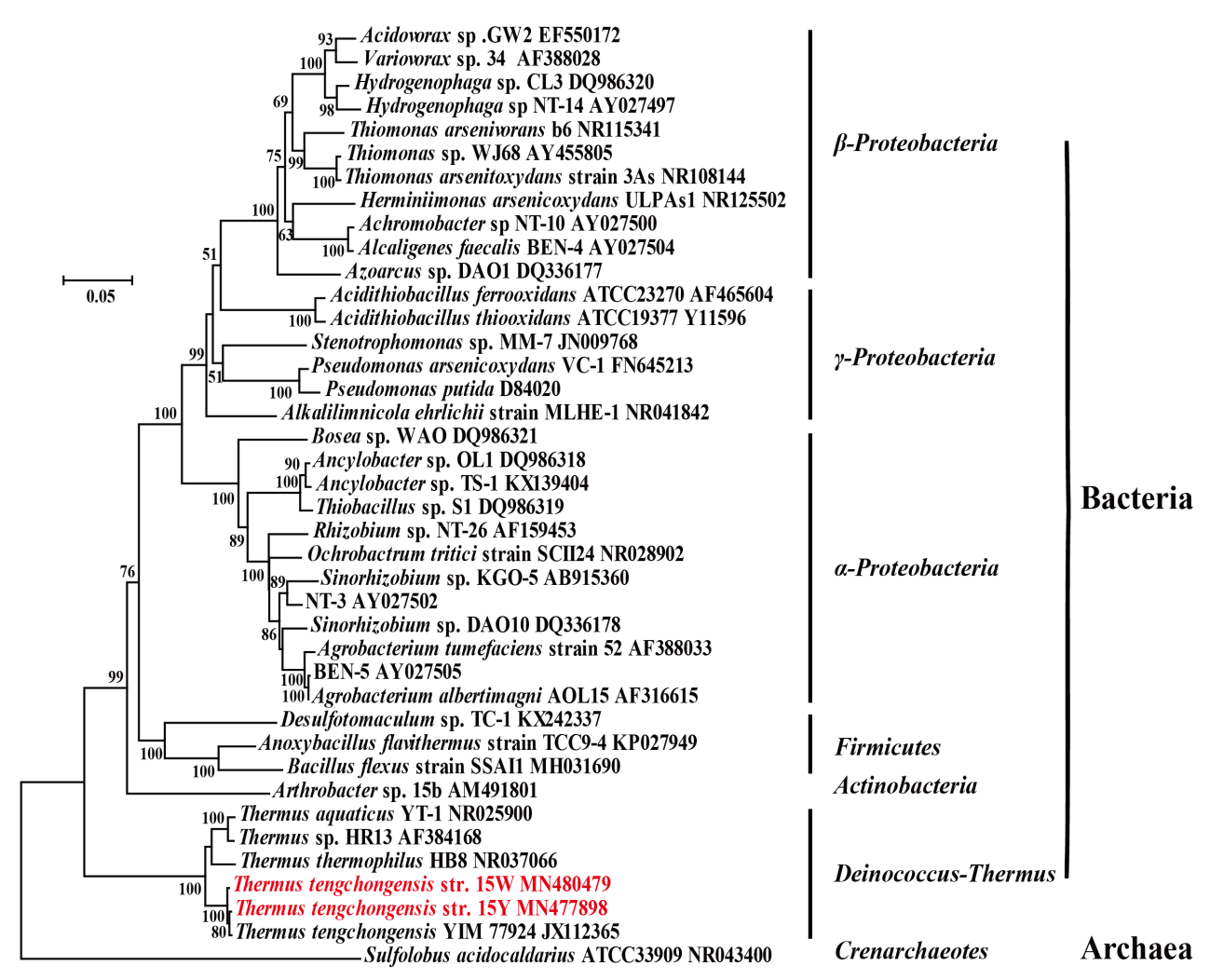
**

**Supplementary Figure 3.** Phylogenetic relationship between two strains and other As(III) oxidizers based on 16S rRNA gene. Outgroup is *Sulfolobus acidocaldarius* ATCC 33909. Stability of relationship was assessed by bootstrap values (shown >50 for 1000 resampling) for tree topology of neighbor-joining data. Bars at the bottom of the tree denote the percentage of substitutions.

**
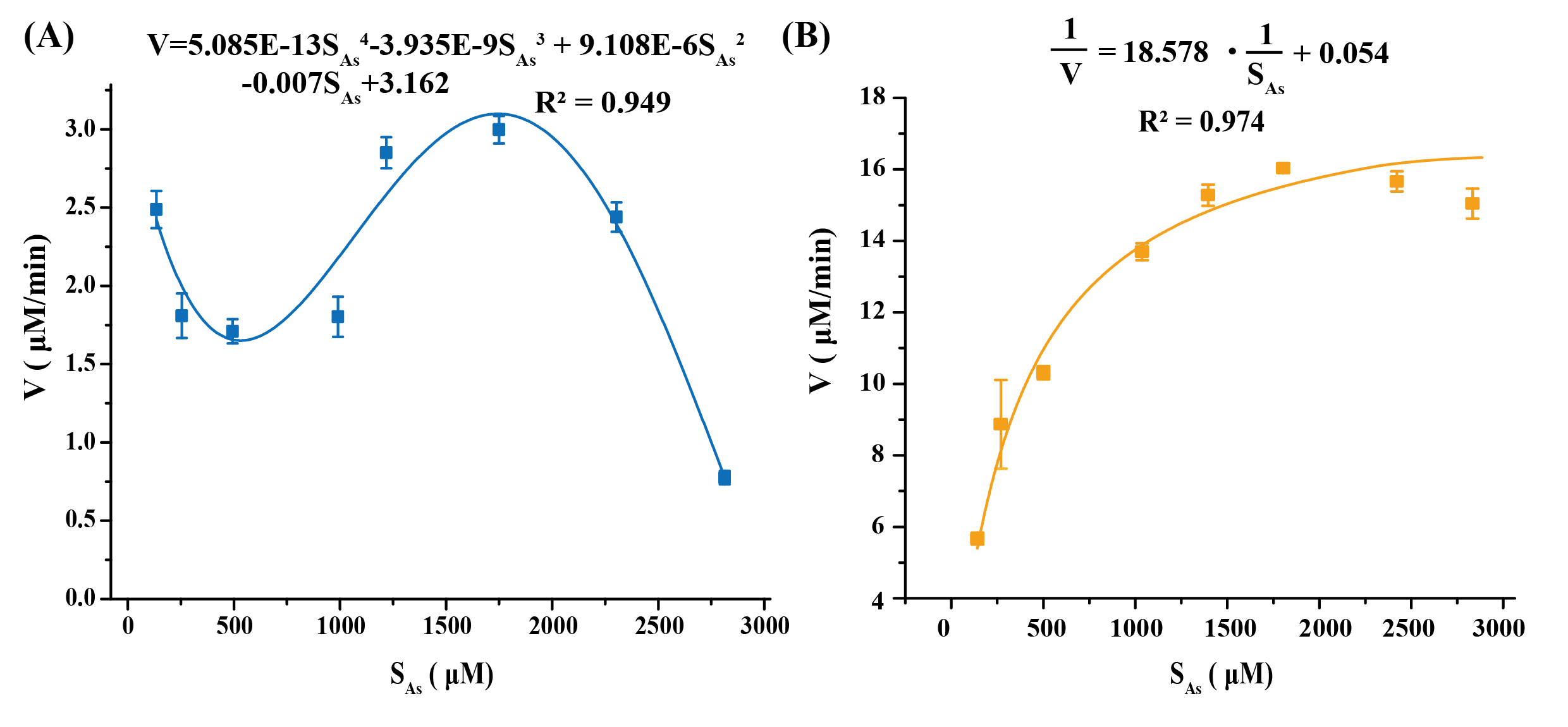
**

**Supplementary Figure 4.** Velocity versus substrate concentration depicting possible kinetic models of strains 15Y (A) and 15W (B). X-axis represents the As[III] concentration and Y-axis stands for oxidation rate. The incubation time interval with is 25 min.

**
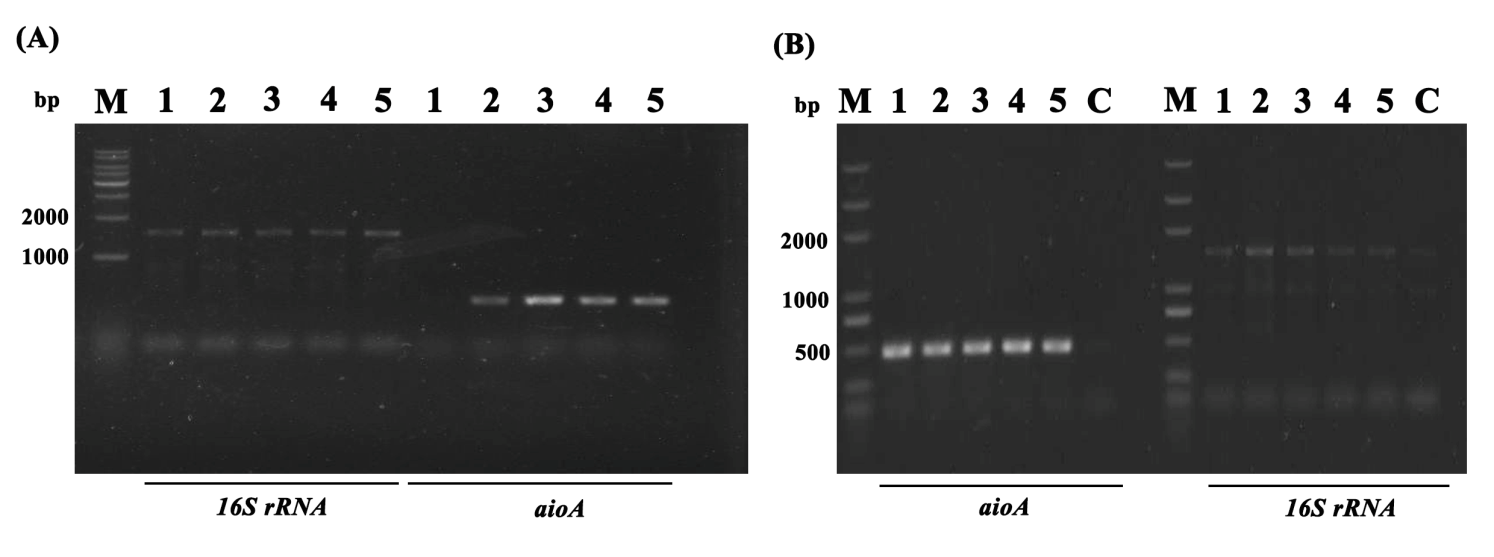
**

**Supplementary Figure 5.** Agarose gel visualization of RT-PCR of *aioA* and 16S rRNA genes of strains 15Y and 15W. Their product sizes are about 0.5 and 1.5kp, respectively. cDNA products derived from total RNA between strain 15Y (A) and strain 15W (B). Lane 1-5 was samples without As[III], treated with As[III] for 0.5 h, 2h, 4h, 6h, respectively. Lanes C were controls without reverse transcriptase confirming the remove of genomic DNA.

**Supplementary Table 1.** Genome characteristics and comparison between the genomes of strain 15Y and strain 15W with genome of the closest type strain of *Thermus tengchongensis* from hot spring

| **Species name** | **Strain. No.** | **WGS accession** | **BioSample** | **BioProject** | **Genome size (bp)** | **Contigs** | **Genome Coverage** | **N50 (bp)** | **Gene number** | **GC content (%)** | **t**  **RNAs** | **r**  **RNAs** | ***ANIm (%)** | ****Digi**  **-DDH (%)** |
| --- | --- | --- | --- | --- | --- | --- | --- | --- | --- | --- | --- | --- | --- | --- |
| *Thermus tengchongensis* | YIM 77401^T^ | NZ_JQLK00000000 | SAMN02745444 | PRJNA234779 | 2,562,314 | 5 | unknown | unknown | 2726 | 66.4 | 47 | 3 | 100 | 100 |
|  | Strain 15Y | NZ_SKBL00000000 | SAMN11053606 | PRJNA525562 | 2,435,142 | 62 | 240.0x | 105,801 | 2605 | 66.7 | 47 | 3 | 98.55 | 98.16 |
|  | Strain 15W | NZ_SJZF00000000 | SAMN11054000 | PRJNA525592 | 2,439,206 | 66 | 360.0x | 93,976 | 2599 | 66.6 | 47 | 4 | 97.88 | 94.09 |

* Average nucleotide identities between the strain 15Y and strain 15W with the type strain of *Thermus tengchongensis* YIM 77401^T^

** Digital DNA–DNA hybridization (<http://ggdc.dsmz.de/distcalc2.php>) values were calculated with the genome of related *Thermus tengchongensis* YIM 77401^T^*.*

**Supplementary Table 2.** Detail of primer pairs and RT-PCR thermal cycling parameters for 16S rRNA, *recG*, *aioA*, *aioB* genes.

| Target genes | Primer name | Sequence (5′ -3′) of primer pairs | PCR cycling programs |
| --- | --- | --- | --- |
| 16S rRNA | Bac27F  Univ1492R | AGAGTTTGGATCMTGGCTCAG  CGGTTACCTTGTTACGACTT | 95 °C for 5 min; 35 cycles of 30 s at 94 °C, 30 s at 55 °C, 2 min at 72 °C, and 7 min at 72 °C |
| *recG* | recG-F2  recG-R2 | GGATCACAGCCAGAAGACCCTAAAG  TCGCTCAATGATCTCGGTGTCCT | 95 °C for 30 s; 40 cycles of 95 °C for 5 s and 60 °C for 30 s |
| *aioA*^*^ | aioA-F1  aioA-R1  781F  1234R | AAGCCAACGACACCTCCATCAAC  AACTTAACCGCCTCCTGGACCTT  GTAGCGGAGAAGCAGCAA  CCGCCTGGTCGTAGTTCT | 95 °C for 30 s; 40 cycles of 95 °C for 5 s and 60 °C for 30 s  95 °C for 5 min; 35 cycles of 30 s at 94 °C, 30 s at 55 °C, 2 min at 72 °C, and 7 min at 72 °C |
| *aioB* | aioB-F1  aioB-R3 | GCCAGCCCATCATCTTCAACTACCC  CATGGAGTAATGGCACCGGCAGAT | 95 °C for 30 s; 40 cycles of 95 °C for 5 s and 60 °C for 30 s |

^*^aioA-F1/R1 used for quantitative real-time PCR (RT-qPCR) analysis, its product size 168 bp is suitable for RT-qPCR with blue color; 781F/1234R used for reverse transcription-PCR analysis, its product size 450 bp with golden color

**Supplementary Table 3.** The original activity data for As[III] oxidation kinetics of two strain with 25 min interval. 0.2, 0.4, 0.8, 1.6, 2, 3, 4, 5 μl of the 500 mM stock solution of As[III] was added in 1ml fresh medium with 10^7^ CFU mL^-1^, respectively. Each As(III) concentration has three replicates, and CK stands for the absence of cell.

| As (μM) | 15Y | | | 15W | | | CK | | |
| --- | --- | --- | --- | --- | --- | --- | --- | --- | --- |
| As[III] | 75.12 | 68.04 | 73.25 | 0 | 0 | 0 | 150.55 | 147.35 | 152.14 |
|  | 204.71 | 210.81 | 210.58 | 68.47 | 21.71 | 55.15 | 295.49 | 299.24 | 303.57 |
|  | 454.78 | 448.87 | 448.12 | 244.71 | 252.86 | 229.35 | 437.64 | 425.84 | 433.26 |
|  | 940.81 | 946.71 | 948.67 | 690.87 | 701.49 | 689.36 | 1080.89 | 1105.64 | 1098.25 |
|  | 1136.87 | 1153.71 | 1148.46 | 1011.92 | 1007.24 | 1023.14 | 1341.40 | 1315.85 | 1338.34 |
|  | 1659.55 | 1691.38 | 1671.71 | 1405.14 | 1394.56 | 1408.89 | 1613.52 | 1621.54 | 1615.72 |
|  | 2255.48 | 2160.18 | 2312.09 | 2047.08 | 2017.51 | 2026.84 | 2184.61 | 2204.83 | 2191.47 |
|  | 2827.20 | 2794.34 | 2760.28 | 2417.24 | 2438.49 | 2432.37 | 2761.64 | 2813.45 | 2803.24 |
| As[V] | 62.18 | 59.24 | 65.18 | 141.32 | 137.84 | 146.18 | 0 | 0 | 0 |
|  | 42.25 | 49.18 | 44.28 | 206.17 | 257.61 | 201.57 | 0 | 0 | 0 |
|  | 42.08 | 41.21 | 44.92 | 257.24 | 262.67 | 253.29 | 0 | 0 | 0 |
|  | 48.62 | 44.28 | 42.37 | 339.61 | 349.27 | 338.39 | 0 | 0 | 0 |
|  | 68.64 | 71.64 | 73.54 | 382.17 | 389.34 | 374.51 | 0 | 0 | 0 |
|  | 75.71 | 72.46 | 76.67 | 403.75 | 397.52 | 401.15 | 0 | 0 | 0 |
|  | 61.78 | 58.36 | 62.82 | 392.27 | 384.26 | 398.36 | 0 | 0 | 0 |
|  | 20.44 | 18.28 | 19.36 | 386.27 | 371.64 | 365.92 | 0 | 0 | 0 |
